# Hydraulic Response of Deciduous and Evergreen Broadleaved Shrubs, Grown on Olympus Mountain in Greece, to Vapour Pressure Deficit

**DOI:** 10.3390/plants11081013

**Published:** 2022-04-08

**Authors:** Maria Karatassiou, Panagiota Karaiskou, Eleni Verykouki, Sophia Rhizopoulou

**Affiliations:** 1 Laboratory of Rangeland Ecology, School of Forestry and Natural Environment, Aristotle University of Thessaloniki, 54124 Thessaloniki, Greece; giotkara81@gmail.com; 2 Department of Agriculture, Crop Production and Rural Environment, University of Thessaly, Fytokou St., 38446 Volos, Greece; everykouki@uth.gr; 3 Section of Botany, Department of Biology, National and Kapodistrian University of Athens, 15784 Athens, Greece; srhizop@biol.uoa.gr

**Keywords:** water potential, stomatal conductance, transpiration, leaf hydraulic conductance, drought

## Abstract

In this study, leaf hydraulic functionality of co-occurring evergreen and deciduous shrubs, grown on Olympus Mountain, has been compared. Four evergreen species (*Arbutus andrachne*, *Arbutus unedo*, *Quercus ilex* and *Quercus coccifera*) and four deciduous species (*Carpinus betulus*, *Cercis siliquastrum*, *Coronilla emeroides* and *Pistacia terebinthus*) were selected for this study. Predawn and midday leaf water potential, transpiration, stomatal conductance, leaf temperature and leaf hydraulic conductance were estimated during the summer period. The results demonstrate different hydraulic tactics between the deciduous and evergreen shrubs. Higher hydraulic conductance and lower stomatal conductance were obtained in deciduous plants compared to the evergreens. Additionally, positive correlations were detected between water potential and transpiration in the deciduous shrubs. The seasonal leaf hydraulic conductance declined in both deciduous and evergreens under conditions of elevated vapor pressure deficit during the summer; however, at midday, leaf water potential reached comparable low values, but the deciduous shrubs exhibited higher hydraulic conductance compared to the evergreens. It seems likely that hydraulic traits of the coexisting evergreen and deciduous plants indicate water spending and saving tactics, respectively; this may also represent a limit to drought tolerance of these species grown in a natural environment, which is expected to be affected by global warming.

## 1. Introduction

The climate in the Mediterranean region is characterized by a prolonged summer drought period, which according to the majority of climate change scenarios, is expected to become more severe in the future [1,2,3]. Drought is the main environmental stress directly linked to plant survival, growth, competitiveness, persistence, and productivity in Mediterranean habitats that are under an increasing risk of degradation [1,4,5,6,7]. On the other hand, plants have evolved a variety of morphological and physiological tactics [8] to withstand drought stress [9,10]; these include (a) hydraulic strategies via deep, tap root systems to extract water from deep soil layers, enabling the plants to sustain elevated water potential and xylem pressure during drought and (b) morphological and physiological adaptations at the leaf level in order to reduce water loss [11,12]. The importance of the hydraulic system in water consumption has led to the hypothesis of the functional convergence in the regulation of water use among phylogenetically diverse species [13,14]. Nonetheless, few studies have been performed to characterize the differences in leaf hydraulic conductance and hydraulic status among phylogenetically different plant species, such as deciduous and evergreen shrubs, in Mediterranean habitats [15,16]. Leaf is a substantial organ for the transport of water throughout the plant and hence leaf hydraulic conductance is an important parameter to determine plant water status [15,17,18,19,20]. To the best of our knowledge, a comparative study among hydraulic characteristics at the leaf level of evergreen and deciduous species co-occurring in the same habitat, with respect to the impact of climatic stimuli on functionality, has not hitherto been published.

In order to comprehensively address differences between evergreen and deciduous plants concerning physiological responses to drought, the hydraulic and stomatal performance should be examined with well-established approaches. The water status for each individual, specific plant depends on the difference between transpiration (Ε) and water absorption (A), but it is not clear which of these factors is more important for plants’ gas exchanges in response to drought stress [12]. If the soil water shortage increases, water stress will increase over time, negatively affecting many physiological and metabolic aspects of the plants [8,21,22]. Plants close the stomatal pores to regulate water loss [12,23] through transpiration when the available soil water decreases and/or an increase in the difference between the saturation (i.e., amount of water vapor that the air can hold, namely the saturation vapor pressure) and actual vapor pressure, i.e., the Vapour Pressure Deficit (VPD), is detected.

VPD has been recognized as an important parameter for plant functionality and survival, which is influenced by the so-called hydraulic failure [24,25,26,27,28,29]. Oren et al. [30] fount in drought tolerant species a regulation of water potential (Ψ) as VPD increases and recommended different sensitivity of stomatal apparatus to VPD among different functional groups. However, Ε could either increase or decrease in response to an increased VPD [27,31]; the first response is known as “feedback” response while the second as “feed forward” response. The transpiration rate is influenced by atmospheric conditions and, over a short time-scale, is regulated by the function of stomatal apparatus [32]. It has been argued that a declining stomatal conductance (g_s_) concomitantly with increasing VPD would rather occur as a feedback response to E and water loss from the leaf, than as a direct response to humidity [33,34,35].

The function of the stomatal apparatus under global climate change is an essential subject in plant ecophysiological research because it affects plant growth, vegetation distribution and ecosystem function [36]. The stomatal response to VPD varies either across and within species, or within the same species [37,38,39,40]. Partial stomatal closure under elevated VPD, especially during midday, will negatively affect CO_2_ assimilation rate [7,41].

The movement of water in the soil–plant–atmosphere (SPA) continuum is a function of the difference between hydrostatic pressure and hydraulic conductance along this pathway. Water flows through the SPA continuum driven by a gradient in Ψ, which depends on the water flow rate and the hydraulic conductivity of the different pathways [32]. The amount of water that will be absorbed by the roots of the plants and the amount that will move from the roots to the leaves and then to the atmosphere depends mainly on g_s_, VPD and hydraulic conductance (K). The differences between plant species in Ψ, E and g_s_, determine to a greater or lesser extent the plants’ response to various water stresses [12,42,43,44,45]. However, the effects of rising VPD on vegetation and hydraulic dynamics remain poorly studied. It has been reported that significant higher leaf and stem hydraulic conductance [25,46,47] under increased irradiance are due to the regulation of aquaporins [46,47,48]. A midday decline of g_s_ has been obtained in many plant species and has been related with variation in midday stem water status [49,50]; this aspect supports the idea that stomatal response to VPD is substantially related to the hydraulic characteristics at both the whole plant scale and the leaf level [27,51]. There is a fundamental role of hydraulics on stomatal sensitivity to VPD [27,41], while inverse and non-linear relationship between conductance and VPD have been observed [36]. The opening and closing of the stomata seem to be controlled by complex mechanisms, which include chemical and hydraulic signals from roots, to shoots and leaves [52]. In this frame, hydraulic traits could be an important factor buffering the negative impact of drought on plant function [53].

A lot of research has been devoted to understanding how plants’ hydraulic systems have evolved to accommodate survival under different environments. However, concerning old-grown, trees and shrubs the relationship between K and the response of g_s_ to VPD has not explicitly evaluated, in situ. Despite the above-mentioned trends, species grown under the same environmental conditions may exhibit entirely different hydraulic properties [54,55]. This interspecific variation sometimes may be ascribed to different functional types, such as deciduous and evergreen [56]. Nevertheless, variation of hydraulic traits cannot only be explained by categorization of species in functional types [56,57]. Forest and shrub communities’ response to climate change are most closely related to microclimatic change and not to macroclimatic change [58,59].

In considering that the water balance (W) is expressed by the relationship W = A − Ε, it is very interesting to study whether: (1) variation of hydraulic conductance in Mediterranean shrubs during the dry summer period is reflected in the leaf phenology (i.e., evergreen vs. deciduous), and (2) changes of VPD and consequently of microclimatic conditions influence the physiological mechanism and the performance of evergreen vs. deciduous shrubs.

The main objective of this study was to compare the ability of co-existing deciduous and evergreen broadleaved shrubs grown on the Olympus Mountain to maintain their water status and control their stomatal conductance throughout the dry season, as well as to evaluate the response of deciduous and evergreen shrubs to enhanced vapor pressure deficit. It is likely that hydraulic responses of co-occurring deciduous and evergreen shrubs, which are to some extent linked to water exploitation and effectiveness of plants’ life forms during a period of soil drying, have not been reported.

## 2. Results

### 2.1. Climatic Conditions

**Figure 1:**
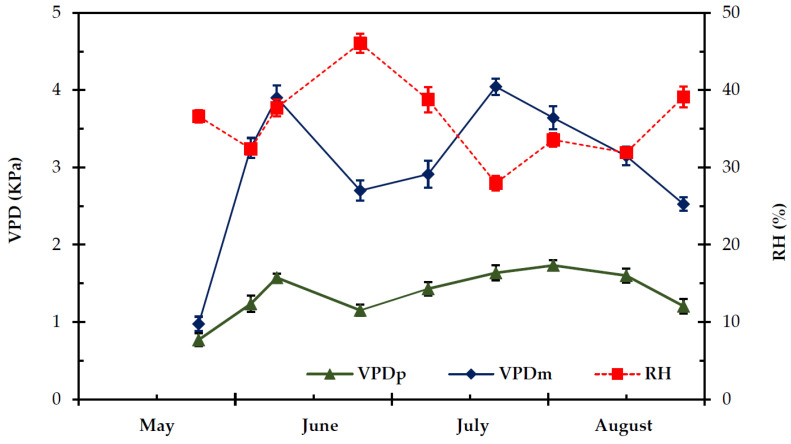
Seasonal predawn (VPD_p_) and midday (VPD_m_) vapour pressure deficit and relative humidity (RH) in the study site, during the experimental period, from 27 May to 28 August. The values are means ± SE (*n* = 24).

**Figure 2:**
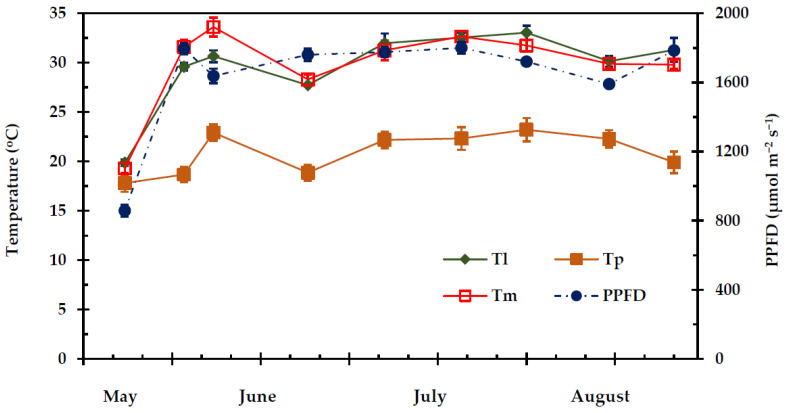
Seasonal Photosynthetic Photon Flux Density (PPFD), leaf temperature (T_l_), morning (T_p_) and midday (T_m_) ambient air temperature during the experimental period from 27 May to 28 August. The values are means ± SE (n_Tp,Tm_ = 24, n_Tl_ = 48).

The seasonal pattern of VPD during the experimental period is given in Figure 1. Overall, predawn vapour pressure deficit (VPD_p_) was significantly lower (*p* < 0.05) than midday vapour pressure deficit (VPD_m_), except from the first measurement (mid-May) (*p* ≥ 0.05). Predawn VPD_p_ ranged from 0.769 ± 0.019 kPa to 1.731 ± 0.054 kPa, while VPD_m_ ranged from 0.974 ± 0.044 kPa to 4.043 ± 0.106 kPa. The relative humidity (RH) followed a reverse course relatively to VPD during the experimental period (Figure 1). The Photosynthetic Photon Flux Density (PPFD) was maintained at a relatively high level (i.e., >1500 μmol m^−2^ s^−1^) (Figure 2) from the end of May to the end of August; only during the first measurement (end of spring) PPFD was relatively low, approximately 800 μmol m^−2^ s^−1^.

Data analysis revealed that only the date was a significant predictor for leaf temperature (T_l_) (*p* < 0.001), whereas the shrub life form (deciduous, evergreen) and the interaction between date and shrub life form were not significant (*p* ≥ 0.05). Leaf temperature (T_l_) during the study period almost coincided with the midday ambient air temperature (T_m_), ranging from 19.86 °C to 33.04 °C and 19.29 °C to 33.61 °C, respectively (Figure 2). Predawn air temperature (T_p_) ranged from 17.80 °C to 23.21 °C. The differences between T_p_ and T_m_ were statistically significant (*p* < 0.05) throughout the experimental period, except from the first measurement (mid-May).

### 2.2. Physiological Parameters

**Figure 3:**
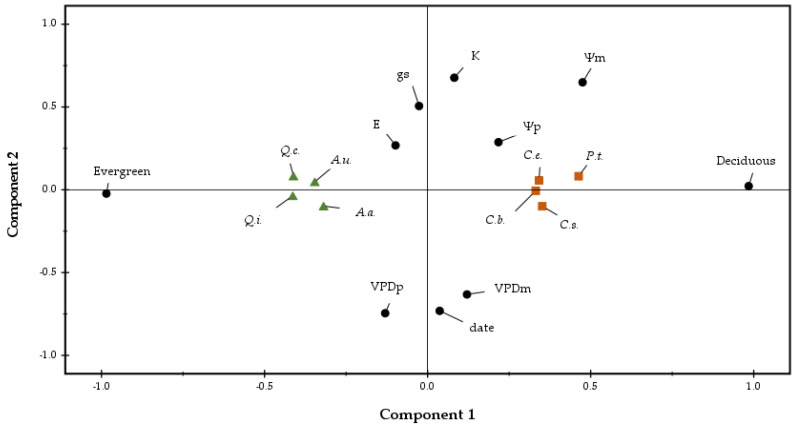
Principal component analysis (PCA) loading plot representing the variables and the species characterizing the two components. Black dots indicated predawn (Ψ_p_) and midday (Ψ_m_) leaf water potential, midday transpiration rate (E), midday stomatal conductance (g_s_), midday leaf hydraulic conductance (K_Leaf_) and midday vapour pressure deficit (VPD_m_); brown squares indicated the deciduous species: *Coronilla emeroides* (*C.e*.), *Carpinus betulus* (*C.b*.), *Pistasia terebinthus* (*C.t.*). *Cercis siliquastrum* (*C.s.*), while green triangles indicated the evergreen species: *Arbutus unedo* (*A.u*.), *Arbutus adrachnae* (*A.a.*), *Quercus coccifera* (*Q.c*.) and *Quercus ilex* (*Q.i.*).

The principal component analysis (PCA, Figure 3) of the physiological parameters showed that the eight species are classified into the groups they belong to, deciduous and evergreen, and characterize the first principal component (Axis x), i.e., the species *Coronilla emeroides* Boiss. and Spruner, *Carpinus betulus* L., *Pistasia terebinthus* L. and *Cercis siliquastrum* L. are deciduous, while the species *Arbutus unedo* L., *Arbutus adrachnae* L., *Quercus coccifera* L. and *Quercus ilex* L. are evergreens. Additionally, variables such as predawn leaf water potential (Ψ_p_), g_s_ and leaf hydraulic conductance (K_Leaf_) are negatively correlated with VPD_p_, VPD_m_, the Julian date of the measurements and the second principal component (Axis y). Midday leaf water potential (Ψ_m_) characterizes both components, while E does not characterize any of the two components. The deciduous species presented higher values (less negative) of Ψ_p_, Ψ_m_, g_s_ and K_Leaf_ relatively to evergreen species (Table 1).

### 2.3. Leaf Water Potential

**Figure 4:**
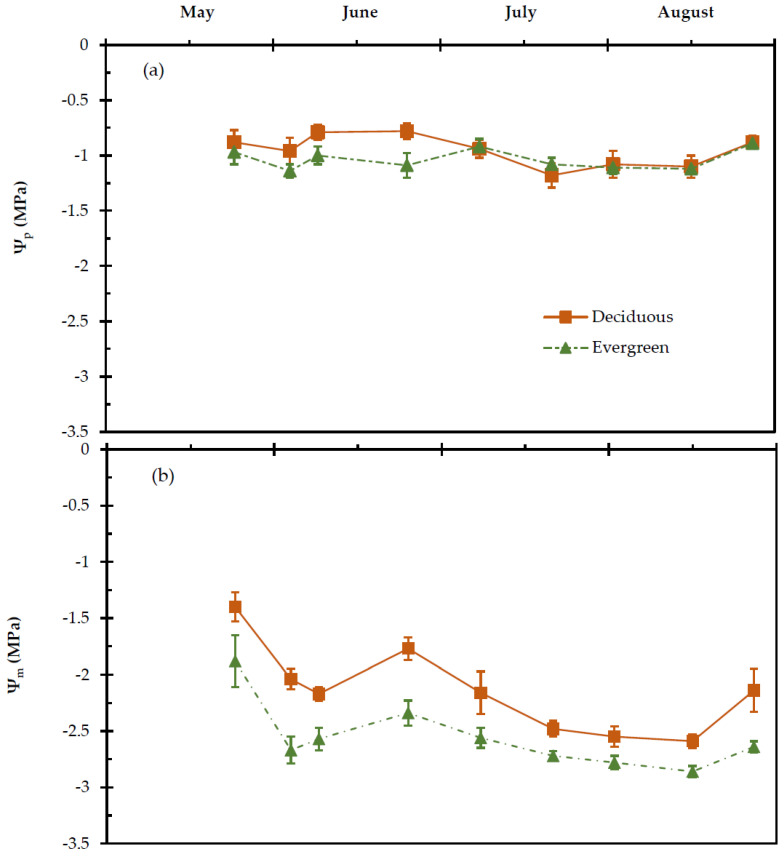
Seasonal (**a**) predawn (Ψ_p_) and (**b**) midday (Ψ_m_) leaf water potential of deciduous and evergreen shrubs during the experimental period from 27 May to 28 August. The values are means ± SE (*n* = 24).

On one hand, data analysis revealed that the date was a significant predictor for Ψ_p_ and Ψ_m_ (*p* = 0.021 and *p* < 0.001, respectively). On the other hand, the shrub life form (deciduous, evergreen) significantly affected the Ψ_m_ (*p* < 0.001). The interaction between date and shrub life form was not significant concerning the two variables (*p* ≥ 0.05) (Figure 4a). The estimated Ψ_m_ values were significantly lower in the considered evergreen shrubs in comparison to the deciduous shrubs during the experimental period (*p* < 0.001), (Figure 4b). Ψ_p_ ranged in deciduous shrubs from –0.87 MPa to −1.21 Mpa and in the evergreens from −0.92 Mpa to −1.14 Mpa, while Ψ_m_ from −1.4 Mpa to −2.59 Mpa and from −1.88 Mpa to −2.86 Mpa, respectively. The average values of the studied parameters are presented in Table 1.

### 2.4. Transpiration Rate, Stomatal and Leaf Hydraulic Conductance

**Figure 5:**
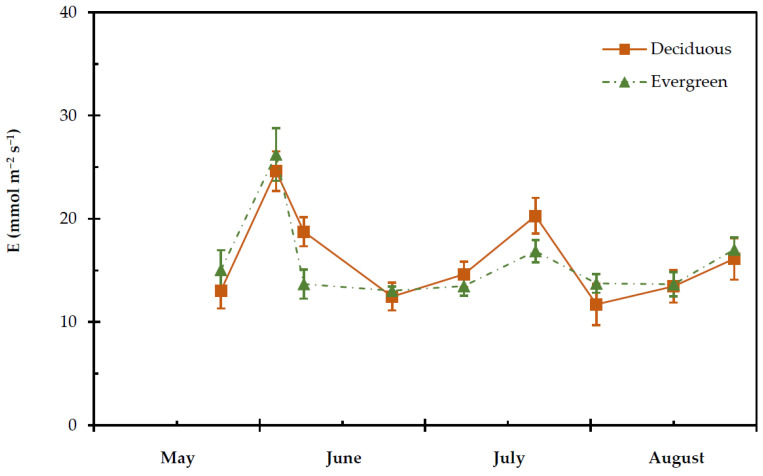
Seasonal transpiration rate (Ε) of deciduous and evergreen shrubs during the experimental period, from 27 May to 28 August. The values are means ± SE (*n* = 24).

Data analysis revealed that the date was a significant (*p* < 0.001) predictor for E, g_s_ and K_Leaf_. The shrub life form (deciduous, evergreen) significantly affected variable K_Leaf_ (*p* < 0.001). The interaction between date and shrub life form was significant only for g_s_ (*p* < 0.05). The seasonal pattern of E (at solar noon) of evergreen and deciduous shrubs did not fluctuate substantially during the experimental period and significant difference was not observed in E between deciduous and evergreen shrubs (*p* > 0.05). The average values of E were found 16.12 ± 0.89 and 15.87 ± 0.68 mmol m^−2^ s^−1^ for deciduous and evergreen shrubs, respectively (Table 1). In particular during the dry season, E ranged from 11.71 to 20.29 mmol m^−2^ s^−1^ in the deciduous and from 13.04 to 17.02 mmol m^−2^ s^−1^ in the evergreen shrubs (Figure 5).

**Figure 6:**
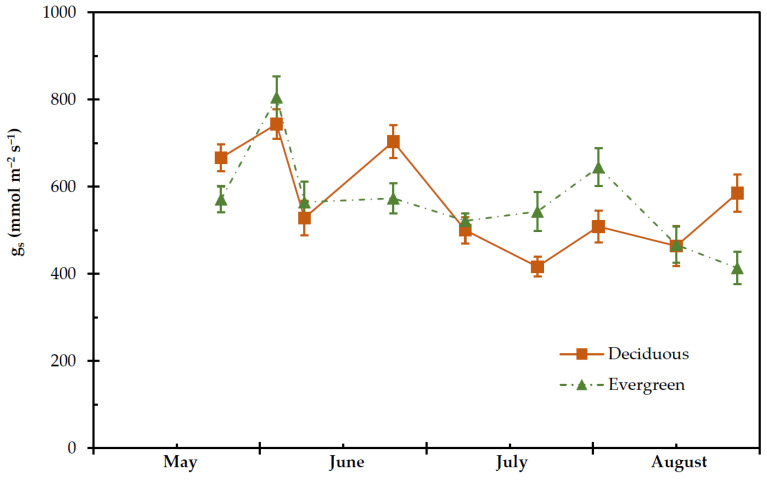
Seasonal stomatal conductance (g_s_) of deciduous and evergreen shrubs during the experimental period, from 27 May to 28 August. The values are means ± SE (*n* = 24).

Seasonal pattern of g_s_ was different (*p* < 0.05) between the two groups of plants. The deciduous shrubs presented significantly lower values (*p* < 0.05) of g_s_ in relation to the evergreens during dry period (from July until mid-August). The g_s_ during dry period ranged from 416.5± 22.7 to 585.25 ± 42.5 mmol m^−2^ s^−1^ in deciduous and from 413.5 ± 27.4 to 644.75 ± 43.5 mmol m^−2^ s^−1^ in evergreen shrubs (Figure 6).

**Figure 7:**
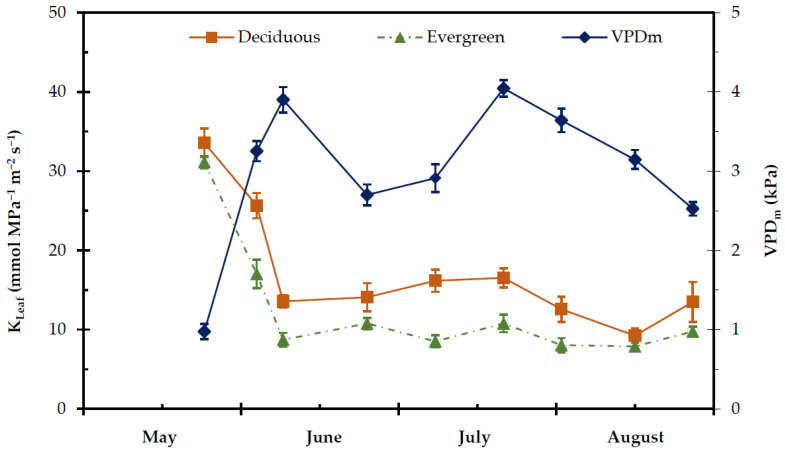
Seasonal midday vapour pressure deficit (VPD_m_) and leaf hydraulic conductance (K_Leaf_) of the considered deciduous and evergreen shrubs during the experimental period, from 27 May to 28 August. Values are means ± SE (n_Kleaf_ = 24, n_VPDm_ = 48).

The K_Leaf_ was significantly higher in deciduous compared to evergreen shrubs (*p* < 0.001), (Figure 7), especially during the dry season. The mean K_Leaf_ was significantly higher in deciduous 17.18 ± 1.45 mmol Mpa^−1^ m^−2^ s^−1^ compared to evergreens 13.15 ± 1.57 mmol Mpa^−1^ m^−2^ s^−1^ (*p* < 0.001), (Figure 7, Table 1). However, the seasonal changes of K_Leaf_ in both groups of plants revealed a decrease in the K_Leaf_ when VPD_m_ increased during the dry period (Figure 7).

**Figure 8:**
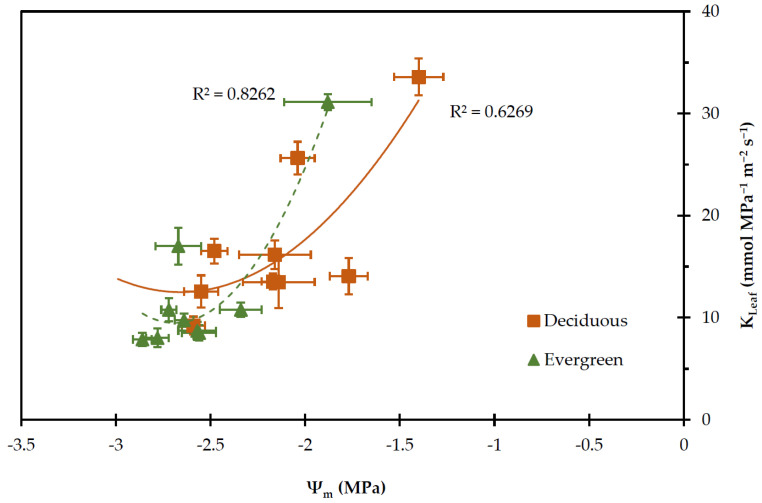
The relationship between midday leaf water potential (Ψ_m_) and hydraulic conductance (K_Leaf_) of deciduous and evergreen shrubs during the experimental period, from 27 May to 28 August. Values are means ± SE (*n* = 24). Polynomial regression analysis was used to assess the relationship between K_Leaf_ and Ψ_m_.

The relationship between K_Leaf_ and Ψ_m_ is presented in Figure 8. It is likely that as the growing season proceeded K_Leaf_ decreased and exhibited the lowest values concomitantly with the lowest Ψ_m_ values. It is worth mentioning that for the same low values of Ψ_m_, the deciduous shrubs exhibited higher K_Leaf_ in relations to the evergreens.

In Table 2, the Pearson correlation between physiological parameters and VPD_m_ is presented. In deciduous species a significant positive correlation was detected between VPD_m_ and E and a negative between Ψ_m_ and g_s_, while in evergreen shrubs VPD_m_ was negatively correlated with Ψ_m_ and K_Leaf_.

## 3. Discussion

The results of this study demonstrate that the water status of co-occurring evergreen and deciduous broadleaved shrubs in semi-arid Mediterranean conditions under rising VPD_m_ is different. Additionally, it seems likely that deciduous shrubs control more efficiently their water status during the dry season, by exhibiting lower stomatal conductance and higher hydraulic conductance than the evergreens. In other words, the deciduous plants possess a more water spending behaviour than the evergreens.

The increase of air temperature accompanied by decreasing RH and increasing heat load due to incident radiation on the leaf, in combination with air speed, may elevate VPD in the atmosphere [60]. It has been reported that RH concerning future climatic scenarios will remain either constant at the global scale [61], or a negative [62] and/or positive trend between VPD and RH at a regional scale [27] will be observed. In our research, the values of PPFD were generally maintained at high levels, as it was expected concerning the characteristics of the Mediterranean climate in the experimental site. The same values in air and leaf temperature imply that broadleaved shrubs possess such leaf tissues, where the transpiration flow is not sufficient to reduce leaf temperature in relation to the ambient temperature. The increase of T_m_ and VPD_m_ under drought conditions (after June) may be linked to the physiological performance and survival of deciduous and evergreen shrubs via either reducing g_s_ and gas exchanges (feedforward mechanism), or increasing plant water loss (feedback mechanism) [63].

The seasonal patterns of Ψ_p_ and Ψ_m_ suggest that the co-occurring deciduous and evergreen shrubs on Olympus Mountain were able to regulate water loss and ensuring adequate hydration of leaf tissues overnight, even during the dry season, therefore Ψ_p_ was retained to approximately −1.0 MPa; such scale of the Ψ_p_ values is sufficient for the Mediterranean region (*Quercetalia ilicis* zone) where the studied species are growing [64]. Ψ_p_ is an essential index to study the response of plants to drought, because its value can be considered equal to soil water potential, since plants and soil reach a hydration equilibrium during the night [65,66,67,68].

The higher (less negative) Ψ_m_ in deciduous shrubs indicates higher values of relative water content, E and g_s_ in comparison to the evergreen shrubs. The Ψ_m_ seems to be the main factors for stomatal regulation because is directly linked to the turgor of guard cells [49,69,70]. After sunrise, the leaf water potential in Mediterranean plants decreases steadily, reaching its lower value at noon, while it begins to recover again during the afternoon [71,72,73]. This reflects a decline of stored water, and therefore water shortage [60]. The plants are the best indicators of soil water availability, which affect their water status [74,75]. Plants with both elevated (less negative) Ψ_m_ and water transport capacity can better control their leaf water status during midday, and hence they experience a smaller decline of midday g_s_ [49].

It is likely that the relatively higher E in deciduous species during midday is due to their elevated water status, i.e., higher Ψ_m_ in leaves (Figure 4a and Figure 5); a slight variation of E values was detected between deciduous and evergreen shrubs, while the lowest values of E occurred in the two groups of shrubs during the dry season (June–August), (Figure 5). Apparently, low E values during the dry season remained rather stable and above zero values, which indicates influx of carbon dioxide [49,76].

The deciduous shrubs exhibited higher Ψ_m_ and lower midday g_s_ than the evergreens; this may indicate that midday stomatal conductance is more related to stem rather than leaf water status, which is in accordance with earlier results [49]. In fact, g_s_ was negatively correlated with VPD_m_ (Table 2), suggesting a response to environmental conditions, which is in agreement with the published results by Auge et al. [77] from other deciduous species. This trait may help deciduous species to regulate their stomatal apparatus in order to maintained Ψ_m_ in a range of values that will support their water status and avoid xylem embolism under drought conditions [49,78]. Our results show that there was not any synchronization between g_s_ and Ψ_m_ in both shrub groups, which may also be due to the fact the evergreens are hypostomatic plants [79,80,81]. However, it is not clear which microclimate parameter, i.e., temperature and/or relative humidity, was directly linked to stomatal behaviour. It has been proposed that temperature had a greater impact on stomatal conductance and assimilation rate in the amphistomatic leaves of *Eucalyptus tetrodonta* independently of VPD [82,83]. Addington et al. [35] reported that the stomatal apparatus controls Ψ in a way that the tension on the water column created by decreasing Ψ did not cause extreme xylem cavitation. Several studies suggested that high VPD reduces g_s_, consequently affecting assimilation rate and growth [84,85]. On the contrary, it has been argued that the impact of high VPD on g_s_ may not possess any impact on assimilation rate and growth [86]. Nevertheless, the impact of VPD on g_s_ and/or assimilation rate varies amongst plant species [82]. In addition, the stomatal anatomy and structure affect water loss and carbon assimilation, demonstrating the evolution and adaptations of the plants to environmental conditions [84,87].

Plants in order to grow and survive under water limited conditions evolved mechanisms to control stomatal aperture and xylem water capacity [88]. The differences in water-use strategies might be partially due to various hydraulic properties between the considered groups. The hydrodynamic status of leaf tissue expressed via leaf water potential [89] is determined by two mail functions taking place in the SPA continuum, i.e., transpiration rate and hydraulic conductance. Therefore, the favourable water status in deciduous shrubs could be attributed to the higher values of transpiration rate and/or at the highest values of the hydraulic conductance. The deciduous shrubs exhibited higher values of K_Leaf_ when compared to evergreen shrubs, especially during the dry summer period. This advantage of deciduous shrubs could be attributed to their water status and anatomical features. The positive correlation between K_Leaf_ and Ψ_m_, and E in deciduous shrubs (Table 2) and the negative correlation between K_Leaf_ and VPD_m_ suggest that the water transport from root to the leaf plays a role to the fluctuations of leaf water status. The seasonal K_Leaf_ declined in both groups of plants when VPD increased during the summer dry period (Figure 7) especially at the end of July, when the highest value of VPD (3.99 KPa) was recorded. Probably, at a given transpiration rate in deciduous shrubs the leaf water status is maintained due to high hydraulic conductance [17,90,91]. Manzoni et al. [88] reported that, in some ecosystems, deciduous species have been found to be more hydraulically efficient than evergreen species. Choat et al. [92] suggested that deciduous species are more hydraulically efficient, but also more vulnerable to drought-induced embolism, than co-existing evergreens in a rainforest. It is well known that embolism occurs in plants under drought conditions when Ψ reaches very low values; however, the plants have the capacity to repair damage when the environmental conditions are favourable [89].

Our data are somehow in agreement with BIumler [93], who argued that although the Mediterranean climate is associated with evergreen species, some coexisting deciduous species exhibit some advantages in response to drought [92,94]. It is noteworthy that in our work deciduous and evergreen species are presented as two distinct life-history groups [91], although they probably form a continuum of variation in leaf life-history span [95].

## 4. Materials and Methods

### 4.1. Study Area and Climate

**Figure 9:**
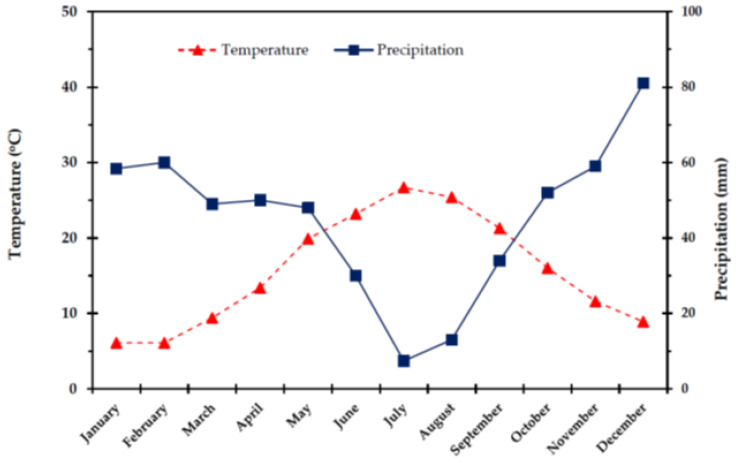
Ombrothermic diagram in the study area throughout a year.

The study was conducted on Olympus Mountain (40°06′54″ N, 22°28′42″ E), which is a great, long-lived natural laboratory [96,97,98] in 2009, at an altitude of 554 m a.s.l., in an area located 5 Km from the town of Litochoro, 95 km south-east of Thessaloniki, in Greece. The climate of the study area is characterized as Cfa in the Köppen-Geiger system (http://www.en.climate-data.org, 7 January 2022) and as Mediterranean with cold and wet winters, dry and warm summer according to the bioclimatogram of Emberger. The mean annual rainfall in the study area ranges from 800 to 1000 mm, while substantially elevated precipitation is recorded during winter. The minimum air temperature ranges from 13 to 6 °C (during summer and winter, respectively). The maximum air temperature ranges from 4.9 to 6.7 °C during winter, and from 20 to 26 °C during summer. The warmest month is July and the coldest is December. The mean annual relative humidity (RH) ranges from 75 to 80%. Average RH values during the most humid month (December) is 85–90% and during the driest month (July) 30–50%. The monthly changes of temperature and precipitation (ombrothermic diagram) during the year of the measurements, from the nearest meteorological station of Dion (40°12′00″ N, 22°30′00″ E), are presented in Figure 9.

The microclimatic conditions (temperature, humidity), in the study area during the experimental period was measured with the Hobo H8 Pro (Hobo H8 Pro Series 1997–2003, Onset Computer Coorporation, Bourne, MA, USA). Furthermore, on specific Julian dates and hours when plant physiological parameters were investigated, ambient air predawn and midday temperature and RH were measured using a Novasima MS1 microclimatic sensor (Novatron Scientific Ltd., Horsham, UK). Predawn (VPD_p_) and midday (VPD_m_) vapour pressure deficit were calculated according to Abtew and Melesse (2013). The Photosynthetic Photon Flux Density was measured with the steady-state diffusion porometer LI-1600 (LI- COR, Inc., Lincoln, NE, USA). The presented values of VPD_p_, VPD_m_, RH, PPFD, T_p_ and T_m_ are means of twenty-four measurements.

### 4.2. Plant Material

The study area is part of the Mediterranean zone of the evergreen broadleaved plants *Quercus ilex* L. and *Arbutus andrachne* L. Deciduous and evergreen shrubs and small trees such as *Acer monspesulanum* L., *Carpinus betulus* L., *Cercis siliquastrum* L., *Cotinus coggygria* Scop., *Fraxinus ornus* L., *Pistacia terebinthus* L., *Coronilla emeroides* Boiss. and Spruner, *Quercus coccifera* L., *Arbutus unedo* L., *Phillyrea media* L., and *Juniperus oxycedrus* L. are widespread in the study area.

Four evergreen shrubs *Arbutus andrachne* (commonly known as Grecian strawberry tree), *Arbutus unedo* (strawberry tree), *Quercus ilex* (holm oak) and *Quercus coccifera* (kermes oak) and four deciduous shrubs *Carpinus betulus* (common hornbeam), *Cercis siliquastrum* (Juda’s tree), *Coronilla emeroides* (scorpion senna), and *Pistacia terebinthus* (terebinth) were selected for this study. The shrubs were randomly selected for sampling. More specifically, the area of interest was equal to 20 ha. We divided this area into 20 tiles, 1 ha each, and then we randomly selected 6 tiles where each one of the eight species was randomly chosen. Concerning dendrometric parameters, the considered species possessed the same height (3–4 m) and diameter (1.5–2.0 m).

### 4.3. Physiological Parameters

Leaf water potential (Ψ), transpiration rate (Ε), stomatal conductance (g_s_) and leaf temperature (T_l_) were measured on the newest fully developed mature leaves of six different individuals from sun-exposed terminal branches. Using the pressure-bomb technique (PMS, Albany, OR, USA), leaf water potential was measured twice during the day, i.e., at predawn (Ψ_p_) and midday (Ψ_m_). Transpiration rate, g_s_ and T_l_ were measured using steady state porometer (Li1600, LI-COR Lincoln, NE, USA). Seasonal measurements of Ψ_p_ were obtained before sunrise, while the measurements of Ψ_m_, E, and g_s_ were obtained on clear sunny days at around solar noon (12:00–14:30 h), approximately 10–15 days intervals. The presented values, for each of the parameters, are means of six replications per studied shrub.

Leaf hydraulic conductance (K_Leaf_) was calculating according to Ohm’s law following the formula:

(1) $$K_{\mathrm{Leaf}}=\frac{E}{\left(\Psi_{\mathrm{soil}}-\Psi_{\mathrm{Leaf}}\right)}=\frac{E}{\left(\Psi_{p}-\Psi_{m}\right)}$$

It has been assumed that Ψ_soil_ is in equilibrium with Ψ_p_ and the lowest diurnal Ψ_leaf_ is equal to Ψ_m_ [66,67,99]. However, sometimes the first assumption may lead to overestimation of K_plant_ [100]. Additionally, values of K_Leaf_ reflect the capacity of evergreen and deciduous plants, grown under ambient conditions, for water exploitation during a period of soil drying.

### 4.4. Statistical Analysis

The Kolmogorov–Smirnov test was employed to evaluate data normality. To explore whether the ecophysiological response of deciduous and evergreen shrubs vary during the dry season, a two-way analysis of variance (ANOVA) was performed on the studied parameters (T_L_, Ψ_p_, Ψ_m_, E, g_s_, K_leaf_) [81]. Student’s *t*-test for independent samples was used to examine differences in T_L_, E, g_s_, Ψ_m_, Ψ_p_ and K_Leaf_ between deciduous and evergreen shrubs. The climatic parameters VPD_p_, VPD_m,_ T_p_, and T_m_ at each sampling date were compared using also the Student’s *t*-test for independent samples. Polynomial regression analysis was used to determine the relationship between Κ_Leaf_ and Ψ_m_, between deciduous and evergreen shrubs. In addition, a Principal Component Analysis (PCA) with varimax rotation was used to assess the relationships among the measurements of interest between the eight shrubs to see whether they could be classified according to their ecophysiological response in the life form in which belong (deciduous, evergreen). Pearson correlation was used to explore links among VPD_m_, E, g_s_, Ψ_p,_ Ψ_m_ and K_Leaf_. P-values lower than 0.05 were considered statistically significant. All statistical analyses were performed using the SPSS statistical package v. 27.0 (IBM Corp. in Armonk, NY, USA).

## 5. Conclusions

The results of this research work demonstrate different hydraulic strategies between co-occurring deciduous and evergreen shrubs grown on Olympus Mountain. The deciduous life-form presented a strategy of higher hydraulic and lower stomatal conductance, while the evergreen exhibited lower hydraulic and higher stomatal conductance. Although, seasonal leaf hydraulic conductance declined in both groups of plants when vapor pressure deficit increased during the summer dry period, evergreen shrubs sustain a water transport to their foliage at a rate sufficient to prevent severe damage due to desiccation.

## Figures and Tables

**Table 1:** Mean values of predawn (Ψ_p_) and midday (Ψ_m_) leaf water potential, midday transpiration rate (E), midday stomatal conductance (g_s_), midday leaf hydraulic conductance (K_Leaf_) and midday vapour pressure deficit (VPD_m_) in deciduous and evergreen shrubs during the study period.

Parameter	Deciduous	Evergreens	*p*-Value
	Mean ± SE	Mean ± SE	
Ψ_p_ (MPa)	−0.94 ± 0.04	−1.04 ± 0.03	0.876 ^NS^
Ψ_m_ (MPa)	−2.09 ± 0.06	−2.53 ± 0.05	0.001 **
E (mmol m^−2^ s^−1^)	16.12 ± 0.89	15.87 ± 0.68	0.734 ^NS^
g_s_ (mmol m^−2^ s^−1^)	575.97 ± 21.00	585.5 ± 27.93	0.487 ^NS^
K_Leaf_ (mmol MPa^−1^ m^−2^ s^−1^)	17.18 ± 1.45	13.15 ± 1.57	0.033 *
VPD_m_ (kPa)	2.79 ± 0.13	2.67± 0.11	0.149

* Significant for *p* < 0.05, ** significant for *p* < 0.001, NS—nο significance. Μean values were compared with independent samples Student’s *t*-test.

**Table 2:** Pearson correlation between predawn (Ψ_p_) and midday leaf water potential (Ψ_m_), transpiration rate (E), stomatal conductance (g_s_), leaf hydraulic conductance (K_Leaf_) and midday Vapour Pressure Deficit (VPD_m_) for deciduous (left) and evergreen (right) shrubs.

Deciduous	Ψ_p_	Ψ_m_	E	g_s_	K_Leaf_	VPD_m_	Evergreens
Ψ_p_	1	0.387 **	0.020	−0.139	−0.039	−0.108	Ψ_p_
Ψ_m_	0.524 **	1	−0.311 **	−0.162	0.544 **	−0.451 **	Ψ_m_
E	0.234 *	−0.013	1	−0.328 **	0.135	0.147	E
g_s_	0.357 **	0.620 **	−0.110	1	−0.037	0.157	g_s_
K_Leaf_	0.084	0.478 **	0.562 **	0.209	1	−0.298 **	K_Leaf_
VPD_m_	−0.088	−0.443 **	0.271 **	−0.315 **	−0.147	1	VPD_m_

* Significant for *p* < 0.05, ** significant for *p* < 0.001.

## Data Availability

The data presented in this study are available in figures and tables provided in the manuscript.
